# Representation Method for Spectrally Overlapping Signals in Flow Cytometry Based on Fluorescence Pulse Time-Delay Estimation

**DOI:** 10.3390/s16111978

**Published:** 2016-11-23

**Authors:** Wenchang Zhang, Xiaoping Lou, Xiaochen Meng, Lianqing Zhu

**Affiliations:** 1 School of Instrumentation Science & Opto-Electronics Engineering, Hefei University of Technology, Hefei 230009, China; baomuayi007@126.com; 2 Beijing Key Laboratory for Optoelectronic Measurement Technology, Beijing Information Science & Technology University, Beijing 100101, China; louxiaoping@bistu.edu.cn (X.L.); mengxc@bistu.edu.cn (X.M.); 3 Beijing Laboratory for Biomedical Detection Technology and Instrument, Beijing Information Science and Technology University, Beijing 100101, China

**Keywords:** flow cytometry, spectrally overlapping signals, fluorescence lifetime, time-delay estimation, digital signal processing

## Abstract

Flow cytometry is being applied more extensively because of the outstanding advantages of multicolor fluorescence analysis. However, the intensity measurement is susceptible to the nonlinearity of the detection method. Moreover, in multicolor analysis, it is impossible to discriminate between fluorophores that spectrally overlap; this influences the accuracy of the fluorescence pulse signal representation. Here, we focus on spectral overlap in two-color analysis, and assume that the fluorescence follows the single exponential decay model. We overcome these problems by analyzing the influence of the spectral overlap quantitatively, which enables us to propose a method of fluorescence pulse signal representation based on time-delay estimation (between fluorescence and scattered pulse signals). First, the time delays are estimated using a modified chirp Z-transform (MCZT) algorithm and a fine interpolation of the correlation peak (FICP) algorithm. Second, the influence of hardware is removed via calibration, in order to acquire the original fluorescence lifetimes. Finally, modulated signals containing phase shifts associated with these lifetimes are created artificially, using a digital signal processing method, and reference signals are introduced in order to eliminate the influence of spectral overlap. Time-delay estimation simulation and fluorescence signal representation experiments are conducted on fluorescently labeled cells. With taking the potentially overlap of autofluorescence as part of the observed fluorescence spectrum, rather than distinguishing the individual influence, the results show that the calculated lifetimes with spectral overlap can be rectified from 8.28 and 4.86 ns to 8.51 and 4.63 ns, respectively, using the comprehensive approach presented in this work. These values agree well with the lifetimes (8.48 and 4.67 ns) acquired for cells stained with single-color fluorochrome. Further, these results indicate that the influence of spectral overlap can be eliminated effectively. Moreover, modulation, mixing with reference signals, and low-pass filtering are performed with a digital signal processing method, thereby obviating the need for a high-speed analog device and complex circuit system. Finally, the flexibility of the comprehensive method presented in this work is significantly higher than that of existing methods.

## 1. Introduction

Flow cytometry is a cell biology technique that is widely applied in life science research and in the clinic. It is a well-established and powerful high-throughput fluorescence measurement tool that also allows for the sorting and enrichment of cell subpopulations expressing unique fluorescence signatures. The most common readouts in flow cytometry are the forward- and side-scattering properties of particles and the fluorescence intensities of attached probes measured in multiple spectral channels. Owing to the breadth of measurements conducted in both research and clinical settings, flow cytometers that detect up to 18 colors simultaneously are now available. Yet, the emission spectra of many common fluorophores are broad and the larger the number of colors used, the more prevalent the problem of fluorescence spillover. Because of its reliance on intensity-only signals, flow cytometry cannot easily discriminate between fluorophores that spectrally overlap [1,2].

Detecting the fluorescence lifetime, i.e., the average time for which a fluorophore resides in an excited state, is a possible alternative to intensity-based measurements. The fluorescence lifetime is a central quantity of fluorescence spectroscopy and reflects the fluorophore heterogeneity and the dynamics of the interactions in the fluorophore environment. Early demonstration of the time-resolved flow cytometry technique involved frequency-domain measurements performed with complex analog devices [3,4,5]. In the phase method, harmonic excitation is used when the fluorescence is also harmonic, but with a phase delay and demodulation relative to the excitation, from which the lifetime can be computed. The alternative method, i.e., the time-domain or pulsed technique, directly samples the fluorescence decay curves elicited by very short light flashes via single-photon counting or stroboscopic detection [6,7,8,9]. However, both the time- and frequency-domain approaches increase the cost and complexity of a traditional flow cytometer significantly. Moreover, several high-frequency hardware components must be added to a simple flow system for phase-sensitive flow cytometry to be performed. These modifications include high-frequency mixing and filtering steps, when the employed data acquisition system is unable to sample the high-frequency signals [10,11].

Focusing on the case of spectral overlap occurring for two kinds of fluorochromes, in this paper, we demonstrate a new method for representing spectrally overlapping signals using fluorescence lifetime-dependent methodology. In addition, the autofluorescence overlap are fully handled with the emission fluorescence, without distinguishing the separating influence. This approach, referred to herein as “pulse time-delay estimation” (PTDE), employs a method of digital modulation, rather than a complex and high-speed analog circuit, to separate the fluorescence signals observed from fluorophores emitting fluorescence with differing average lifetimes. PTDE is a practical approach to separating cell populations that are stained with spectrally overlapping fluorophores. 

## 2. Theory

### 2.1. Cytometric Pulse Generation and Fluorescence Spectral Overlap

**Figure 1:**
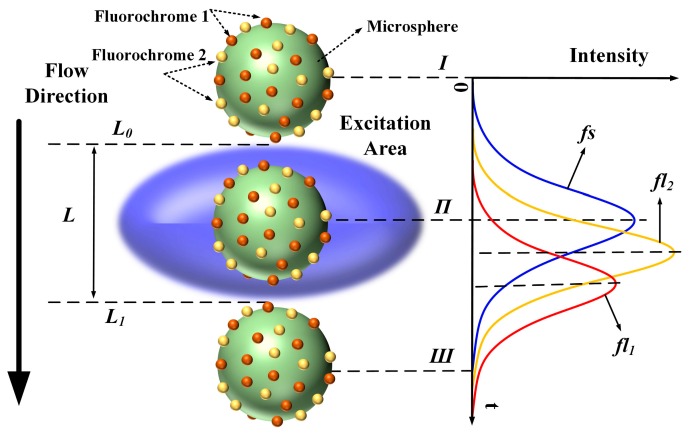
Generation of forward-scattered and fluorescence pulses while microsphere flows through excitation area (*fs*: forward-scattered pulse, *fl*_1_ and *fl*_2_: fluorescence pulses of fluorochromes 1 and 2, respectively; *L*_0_ and *L*_1_: upper and lower limbs of excitation area, respectively).

In flow cytometry, fluorescence signals are often referred to as “cytometric pulses” or “waveforms”, because they are the result of the rapid transit of a fluorescently labeled cell through a tightly focused laser beam with a Gaussian profile. As shown in Figure 1, the microsphere moves along the flow direction through the excitation area (blue ellipsoid, Figure 1). A Gaussian-like trace reflects the resulting signal, where the forward-scattered emission begins to increase at time *Ι* (the microsphere flows across the upper limb *L*_0_ into the excitation area), peaks at time *Π*, and decreases as the microsphere moves from time *Π* to time *Ш* (the microsphere flows across the lower limb *L*_1_).

Therefore, the original signals *fl*_1_ and *fl*_2_ are mathematically expressed as:

(1) $$\{\begin{array}{c} fs=K\exp[-{\left(t-t_{0}\right)}^{2}/(2\sigma^{2})]\\ fl_{1}=A\exp[-{\left(t-t_{0}+\Delta t_{1}\right)}^{2}/(2\sigma^{2})]\\ fl_{2}=B\exp[-{\left(t-t_{0}+\Delta t_{2}\right)}^{2}/(2\sigma^{2})] \end{array}$$

where *fs* is the forward-scattered pulse; *K*, *A*, and *B* are constants representing the heights of the curve peaks; *t*_0_, *t*_0_ − Δ*t*_1_, and *t*_0_ − Δ*t*_2_ represent the peak positions; and σ represents the curve width.

**Figure 2:**
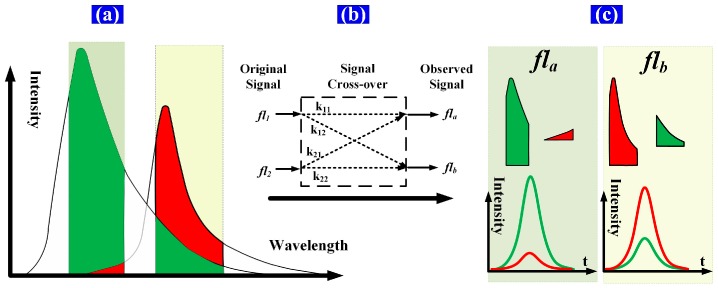
Schematic of spectral overlap of two fluorescence signals. (**a**) The green and red areas represent the spectra of the fluorescence light emitted from fluorochromes 1 and 2, respectively. The spectrum of each fluorochrome is detected by a different detector channel having a fixed bandwidth, and the spectra within the bandwidth of each detector channel are detected as one sample point of the time-intensity cytometric pulse; (**b**) *fl*_1_ and *fl*_2_ represent the original fluorescence signals (time-intensity pulses); *fl_a_* and *fl_b_* represent the observed fluorescence signals after signal crossover; *k*_11_ and *k*_12_ are components of signal *fl*_1_, which contribute to *fl_a_* and *fl_b_*; *k*_21_ and *k*_22_ are components of signal *fl*_2_, which contribute to *fl_a_* and *fl_b_*; (**c**) The green and red pulse (time-intensity cytometric pulse) curves are the respective *fl*_1_ and *fl*_2_ contributions to each detector channel ((*k*_11_ × *fl*_1_, *k*_12_ × *fl*_1_) and (*k*_21_ × *fl*_2_, *k*_22_ × *fl*_2_), respectively).

Spectral overlap, or “spillover”, results from the use of fluorescent dyes that are measurable in more than one detector. This spillover is correlated by a constant known as the spillover coefficient. Spectral overlap is illustrated schematically in Figure 2. Two fluorochromes with different lifetimes are excited simultaneously and then detected using detector channels having fixed bandwidths. The time-intensity Gaussian-like cytometric pulses (*fs*, *fl*_1_, and *fl*_2_) are generated as the microspheres flow through the extinction area. Then, the spectrum of each fluorochrome is detected by a different detector channel having a fixed bandwidth. Both the peak value and the peak location of the detected signal (time-intensity cytometric pulse) of each detector channel are influenced by the spectral overlap due to the different fluorochrome lifetimes. That is, the time delays between the observed fluorescence signals (*fl_a_*, *fl_b_*) and *fs* are influenced by the spectral overlap [12,13,14,15].

In addition, considering the influence of autofluorescence, the dim expression of the exogenous fluorophores adds a number of spectral overlap. Autofluorescence is due to many cellular constituents, rather than a single fluorochrome with a single exponential decay, and it is difficult to distinguish the influence of this behavior from the observed fluorescence spectrum. However, the autofluorescence is dependent on certain cell constituents, it is relatively stable, and its dim intensity barely shifts the peak locations of the fluorescence pulse signals, even with multi-exponential decay. Therefore, the autofluorescence can be fully handled with the emission fluorescence. In what follows, the autofluorescence overlap are regarded as elements of the emission fluorescence for the lifetime calculations of cells labeled with single- or two-color fluorochromes.

**Figure 3:**
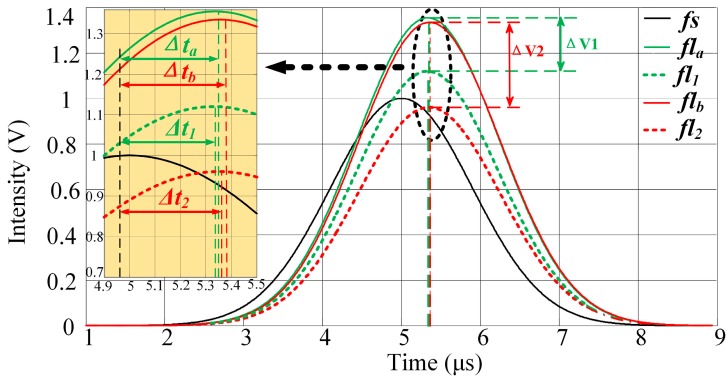
Peak-value errors and pulse time delays introduced by three-signal crossover. Δ*V*_1_ and Δ*V*_2_ represent the differences in the peak values between *fl_a_* and *fl*_1_, and between *fl_b_* and *fl*_2_, respectively. The pulse time-delay details are provided in the yellow region. Δ*t_a_* and Δ*t_b_* are the pulse time delays of *fl_a_* and *fl_b_* (time delays of peak locations between *fs* and *fl_a_*, *fl_b_*) respectively; Δ*t*_1_ and Δ*t*_2_ are the pulse time delays of *fl*_1_ and *fl*_2_ (time delays of peak locations between *fs* and *fl*_1_, *fl*_2_), respectively.

The signals after crossover (*fl_a_*, *fl_b_*) are shown in Figure 3 and described by Equation (2) below. The expected results of the detector channels are *k*_11_*fl*_1_ and *k*_22_*fl*_2_. However, the peak values and peak locations of the observed signals are influenced by signal crossover. The peak values of the observed signals increase with the addition of parts of the crossover signals. The peak-value errors (*Ep_a_*, *Ep_b_*) introduced through crossover can be calculated using Equation (3). Meanwhile, the peak locations of *fl*_1_ and *fl*_2_ differ, and the *fl_a_* and *fl_b_* peak locations move to the left or right when parts of the crossover signals are added. The peak-location errors (*Et_a_*, *Et_b_*) introduced through crossover can be calculated using Equation (4):

(2) $$\{\begin{array}{c} fl_{a}=k_{11}fl_{1}+k_{21}fl_{2}\\ fl_{b}=k_{12}fl_{1}+k_{22}fl_{2} \end{array}$$

(3) $$\{\begin{array}{c} Ep_{a}=\left(P_{a}-k_{11}P_{1}\right)/P_{a}\\ Ep_{b}=\left(P_{b}-k_{22}P_{2}\right)/P_{b} \end{array}$$

Here, *P_a_*, *P*_1_, *P_b_*, and *P*_2_ are the peak values of *fl_a_*, *fl*_1_, *fl_b_*, and *fl*_2_, respectively.

(4) $$\{\begin{array}{c} Et_{a}=\left(\Delta t_{a}-\Delta t_{1}\right)/\Delta t_{a}\\ Et_{b}=\left(\Delta t_{b}-\Delta t_{2}\right)/\Delta t_{b} \end{array}$$

The peak-value errors and the pulse time delay errors (Δ*t_a_* − Δ*t*_1_, Δ*t_b_* − Δ*t*_2_: the time delays of the peak locations between *fs* and the fluorescence pulses (*fl*_1_, *fl*_2_, *fl_a_* and *fl_b_*)) introduced by the signal crossover are shown in Figure 3. It is apparent that Δ*V*_1_ and Δ*V*_2_ are significantly larger than Δ*t_a_* − Δ*t*_1_ and Δ*t_b_* − Δ*t*_2_. Note that the peak values of different fluorescence signals can vary widely and fluorescence intensity measurements are impacted by nonlinearity. Therefore, the peak-value errors introduced by crossover are typically large and are difficult to amend. On the other hand, the differences in the pulse time delays between the different fluorescence pulses are significantly smaller. The pulse time delays vary little with signal crossover. Therefore, the influence of crossover can be decreased be using the pulse time delay as a parameter when representing the fluorescence pulse. 

### 2.2. Fluorescence Pulse Time-Delay Estimation

The time delay between the observed fluorescence signal and the forward-scattered signal includes the lifetime and delay introduced by the hardware [3]. However, only the fluorescence lifetime is relevant for removing the crossover signal from the observed signal in the following sections. Therefore, it is necessary to determine the relationship between the time delay and lifetime. We assume that the fluorescent light is emitted in accordance with the single exponential decay model. The fluorescent-light signal can be expressed as a convolution of the forward-scattered light and the single exponential decay model, such that:

(5) L_fl=K0(L_fs×e−tτ)

where *L_fl* is the fluorescent-light pulse; *L_fs* is the forward-scattered light pulse; *K*_0_ is the signal intensity; and τ is the fluorescence lifetime. Then, we can conclude that the phase shift between *L_fl* and *L_fs* is the phase of e−tτ: arctan(ωτ). Therefore, the time delay between the fluorescent-light pulse and the forward-scattered light pulse is equal to τ. 

To estimate the time delay of output pulse signals from circuit systems, the cross-correlation function algorithm is applied to restrain the influence of the noise. However, the estimation accuracy with a traditional cross-correlation function is limited by the time interval between adjacent points in the pulse signal, i.e., by the sampling frequency of the analog-to-digital converter (ADC). In this work, zoom analysis of the frequency spectrum within the scope of a certain frequency is executed using the modified chirp Z-transform (MCZT) method, so as to acquire the frequency spectral information with high resolution. The frequency resolution is higher with larger *N*_1_, however, more time would be taken for computing. The frequency spectral information is neither limited by the pulse signal length nor the ADC sampling frequency. The fine interpolation of the correlation peak (FICP) algorithm is then used to improve the resolution of the time-domain cross-correlative function. This method avoids modulating the laser at different frequencies and effectively presents a platform for lifetime detection that is easier to practically implement on any standard flow cytometer.

Assuming that the forward-scattered pulse signal *fs*(*n*) and the fluorescence pulse signal *fl_m_*(*n*) are data sequences containing *N* points, the corresponding zoomed frequency spectra (*FS*(*k*) and *FL_m_*(*k*), respectively) are described by the transform:

(6) $$\{\begin{array}{c} FS(k)=MCZT(fs(n))=\sum_{n=0}^{N-1}fs(n)\exp(-\frac{j2\pi }{N_{1}}kn)\\ FL_{m}(k)=MCZT(fl_{m}(n))=\sum_{n=0}^{N-1}fl_{m}(n)\exp(-\frac{j2\pi }{N_{1}}kn) \end{array}$$

where *m* = 1, 2, a, b and *k* = 0, 1, …, *N* − 1.

Based on the sampling theorem, *FS*(*k*) and *FL_m_*(*k*) are periodic functions and the cycle is *N*_1_, while the first and last parts of the functions are conjugate symmetric. With the FICP analysis, the sampling frequency is raised to *N*_2_/*N*_1_ times the initial sampling frequency, when the frequency spectrum cycle is extended from *N*_1_ to *N*_2_ with the insertion of zero. That is, the resolution of the time delay between *fs*(*n*) and *fl_m_*(*n*) in the time domain is increased to *N*_2_/*N*_1_ times for the same number of calculations. The complete cross-correlation frequency spectrum is constructed as:

(7) $$R_{m}(k)=\{\begin{array}{c} R_{1}(k)=FS(k)FL_{m}^{*}(k),\\ 0\\ R_{1}^{*}(N_{2}-k) \end{array}\begin{array}{c} k=0,1,\cdots ,N-1\\ k=N,\cdots ,N_{2}-N\\ k=N_{2}-N+1,\cdots ,N_{2}-1 \end{array}$$

The time delays are distributed within a certain limited range and, similarly, the time delays between *fs*(*n*) and *fl_m_*(*n*) fall into a certain limited range. In this case, only a finite number of the first and last parts of the points are required for the calculation. The first and last parts of the cross-correlation function in the time domain, *r*_1_(*n*) and *r*_2_(*n*), respectively, are calculated from Equations (8) and (9), respectively, as follows:

(8) $$r_{1m}(n)=[\sum_{k=0}^{N-1}R_{1m}(k)\exp(j\frac{2\pi }{N_{2}}kn)+\exp(-j\frac{2\pi }{N_{2}}Nn)\sum_{k=0}^{N-1}R_{1m}^{*}(N-k)\exp(j\frac{2\pi }{N_{2}}kn)]/N_{2}$$

(9) $$\begin{array}{l} r_{2m}(n)=\exp(j\frac{2\pi }{N_{2}}Nn)\sum_{k=0}^{N-1}\left[R_{1m}(k)\exp(-j\frac{2\pi }{N_{2}}kn\right)]/N_{2}\\ \;+\exp[j\frac{2\pi }{N_{2}}(N^{2}-nN)]\sum_{k=0}^{N-1}\left[R_{1m}^{*}(N-k)\exp(-j\frac{2\pi }{N_{2}}kn\right)]\exp(j\frac{2\pi }{N_{2}}kn)/N_{2} \end{array}$$

where *m* = 1, 2, *a*, *b* and *k* = 0, 1, *…*, *N* − 1. *N*_2_ is the length of both *FS*(*k*) and *FL_m_*(*k*) after the zeros are inserted. 

The value of *N*_2_ is set arbitrarily, and the resolution of *r*_1_(*n*) and *r*_2_(*n*) is increased by *N*_2_/*N*_1_. Meanwhile, the integrated cross-correlation function in the time domain *r*(*n*) is constructed with *r*_1_(*n*) and *r*_2_(*n*), such that:

(10) $$r_{m}(n)=\{\begin{array}{cc} r_{1m}(n), & n=0,1,\cdots ,N-1\\ r_{2m}(2N+n) & n=-N,\cdots ,-1 \end{array}$$

The resolution of the cross-correlation peak is improved by increasing the interpolation multiple of the frequency spectrum. That is, the computational accuracy of the time-delay estimation is higher with FICP, particularly for pulse signals of low sampling frequency.

**Figure 4:**
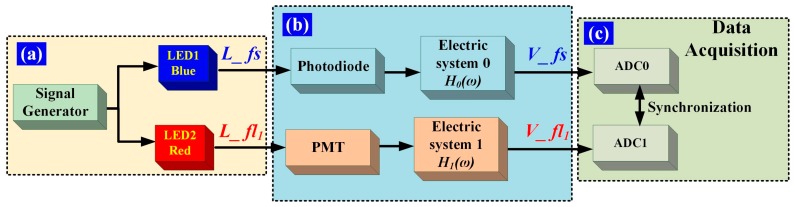
Time-delay calibration. (**a**) Light-pulse generation. The LED1 and LED2 emission lights are blue and red, respectively, and *L_fs* and *L_fl*_1_ are the respective forward-scattered light pulses; (**b**) *L_fs* is detected by a photodiode and processed with electric system 0. *H*_0_(ω) is the transfer function of electric system 0 in the frequency domain. *L_fl*_1_ is detected by a photomultiplier tube (PMT) and processed with electric system 1. *H*_1_(ω) is the transfer function of electric system 1 in the frequency domain. Some light intensity attenuation modules are omitted in the detection of *L_fl*_1_ with a PMT; (**c**) Data acquisition. The analog electrical signals (*V_fs*, *V_fl*_1_) are simultaneously converted to digital signals by ADC0 and ADC1, respectively.

It is necessary to correct for the inherent time delay introduced by the hardware variables, such as the differences in the photoelectric detector, circuitry, and cable. The time delay introduced by the hardware can be eliminated via calibration, which is performed as shown in Figure 4. Two light-emitting diodes (LEDs) are used as light sources and are pulsed synchronously by a standard function generator with Gaussian waveforms. As shown in Figure 4a, the Gaussian-shaped light pulses *L_fs* and *L_fl*_1_ are used to represent the forward-scattered light pulse and the fluorescent-light pulse of a flow cytometer, respectively. *L_fs* and *L_ fl*_1_ are synchronous with the same pulsed signal. Figure 4b shows the photovoltaic conversion and the electric systems. The corresponding electrical pulse signal is presented in terms of *V_fs* and *V_fl*_1_, and then converted to digital signals using ADCs labeled ADC0 and ADC1, respectively, as illustrated in Figure 4c. The ADC0 and ADC1 clock signals are provided by one crystal oscillator, to ensure that *V_fs* and *V_ fl*_1_ are converted simultaneously. The time delay between *V_fs* and *V_fl*_1_ can be acquired and taken as the time delay introduced by the photovoltaic conversion and electric systems.

### 2.3. Representation of Spectrally Overlapping Fluorescence Signals

**Figure 5:**
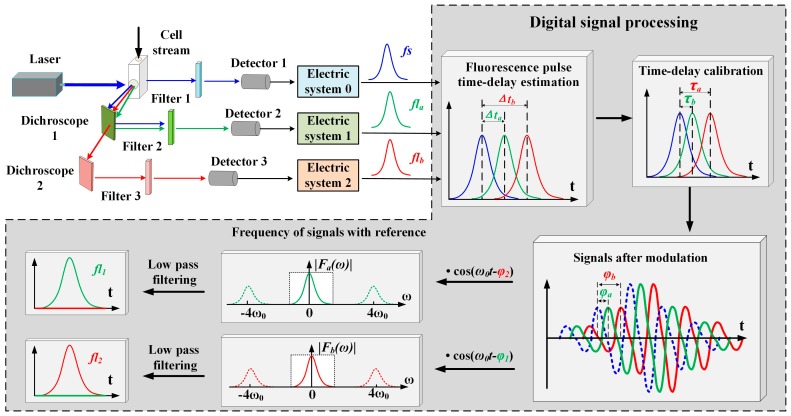
Conceptual diagram of signal detection and processing for PTDE-based fluorescence signal representation. The stages against the dark background are processed using the digital signal processing method. The cell stream is excited by the laser beam in the flow chamber, and the forward-scattered (blue arrows) and fluorescence (green and red arrows) signals from the two fluorochromes are separated with a dichroscope and filter in series. *fs* (blue line) is the forward scattered pulse signal; *fl_a_* and *fl_b_* are the observed summation signals from the first and second channels, respectively; Δ*t_a_* and Δ*t_b_* are the pulse time delays of *fl_a_* and *fl_b_*, respectively; τ_1_ and τ_2_ are the individual lifetime components of fluorochromes 1 and 2, respectively; ϕ_1_ and ϕ_2_ are the phase shifts introduced by τ_1_ and τ_2_, respectively; τ*_a_* and τ*_b_* are the calculated lifetimes of *fl_a_* and *fl_b_*, respectively; and ϕ*_a_* and ϕ*_b_* are the respective phase shifts introduced by τ*_a_* and τ*_b_*.

Spectrally overlapping fluorescence signals are represented using the process shown in Figure 5. The cells are analyzed as they intersect a laser excitation beam. The fluorescence is measured orthogonally to the laser beam-cell stream intersection point in the flow chamber, using a dichroscope, a filter, a signal detector, and an electric system [16]. The time delays (Δ*t_a_*, Δ*t_b_*) of the fluorescence pulses (*fl_a_*, *fl_b_*) are estimated using the above-mentioned FICP arithmetic. As mentioned above, the forward-scattered signal and the fluorescence signal are Gaussian in shape. For a generated signal, *fs*, *fl_a_*, and *fl_b_* can be expressed as shown in Equation (11) [16,17,18].

(11) $$\{\begin{array}{c} fs=K_{0}e^{-\frac{{\left(t-t_{0}\right)}^{2}}{2\sigma^{2}}}=K_{1}e^{-\frac{{\left(t-t_{0}^{'}\right)}^{2}}{2\sigma^{2}}}\times h_{0}(t)\\ fl_{a}=C_{0}e^{-\frac{{\left(t-t_{0}-\Delta t_{a}\right)}^{2}}{2\sigma^{2}}}=C_{1}e^{-\frac{{\left(t-t_{0}^{'}\right)}^{2}}{2\sigma^{2}}}\times e^{-\frac{t}{\tau_{a}}}\times h_{1}(t)\\ fl_{b}=D_{0}e^{-\frac{{\left(t-t_{0}-\Delta t_{b}\right)}^{2}}{2\sigma^{2}}}=D_{1}e^{-\frac{{\left(t-t_{0}^{'}\right)}^{2}}{2\sigma^{2}}}\times e^{-\frac{t}{\tau_{b}}}\times h_{2}(t) \end{array}$$

where, *K*_1_, *C*_1_, and *D*_1_ are the signal intensity, *t*_0_*'* is the center moment of the scattered signal; τ*_a_* and τ*_b_* are the calculated lifetimes of *fl_a_* and *fl_b_*, respectively; and *h*_0_(*t*), *h*_1_(*t*), and *h*_2_(*t*) are the transfer functions of electric systems 0, 1, and 2, respectively.

The individual lifetimes τ_1_ and τ_2_ are acquired so as to express the phase shift introduced by the lifetime. In order to acquire the individual lifetime components of each fluorochrome, the cells are labeled with one kind of fluorochrome only and tested separately. Then, *fl*_1_ and *fl*_2_ are detected, and τ_1_ and τ_2_ are acquired, respectively, using the MCZT and FICP techniques.

The signals after modulation (*fs_mod*, *fl_a__mod*, and *fl_b__mod*) are artificially created using a digital signal processing method, as shown in Equation (12). The modulating signal is a cosine function, and the phase shifts of *fl_a_* and *fl_b_* introduced by τ*_a_* and τ*_b_* are ϕ*_a_* = arctan(ω_0_τ*_a_*) and ϕ*_b_* = arctan(ω_0_τ*_b_*), respectively. Considering the relationship between the observed (*fl_a_*, *fl_b_*) and original signals (*fl*_1_, *fl*_2_), the expressions given in Equation (13) can be acquired. Hence, the phase shifts introduced by τ_1_ and τ_2_ are ϕ_1_ = arctan(ω_0_τ_1_) and ϕ_2_ = arctan(ω_0_τ_2_), respectively.

(12) $$\{\begin{array}{c} fs\_\mathrm{mod}=\cos(\omega_{0}t)K_{2}\exp[-{\left(t-t_{0}\right)}^{2}/(2\sigma^{2})]\\ fl_{a}\_\mathrm{mod}=C_{2}\cos(\omega_{0}t-\varphi_{a})\exp[-{\left(t-t_{0}+\tau_{a}\right)}^{2}/(2\sigma^{2})]\\ fl_{b}\_\mathrm{mod}=D_{2}\cos(\omega_{0}t-\varphi_{b})\exp[-{\left(t-t_{0}+\tau_{b}\right)}^{2}/(2\sigma^{2})] \end{array}$$

(13) $$\{\begin{array}{c} \cos(\omega_{0}t-\varphi_{a})=C_{3}\cos(\omega_{0}t-\varphi_{1})+C_{4}\cos(\omega_{0}t-\varphi_{2})\\ \cos(\omega_{0}t-\varphi_{b})=D_{3}\cos(\omega_{0}t-\varphi_{1})+D_{4}\cos(\omega_{0}t-\varphi_{2}) \end{array}$$

Here, *K*_2_, *C*_2_, *C*_3_, *C*_4_, *D*_2_, *D*_3_, and *D*_4_ are the signal intensities; *t*_0_ is the center moment of *fs_mod*; and ω_0_ is the angular frequency of the modulation.

The aim of PTDE is to transform the observed summation signal, which is measured experimentally, into a signal that is dependent on one fluorophore only. Nullifying one of the fluorescence signals by exploiting the fluorophore lifetime is necessary for this to occur. Nullification is accomplished by mixing a cosine reference signal, which is performed by altering the reference signal phase. The reference signal has the same angular frequency as the modulating signal and the reference phase is shifted by an amount ϕ*_ref_*_1_ *=* ϕ_2_ or ϕ*_ref_*_2_ *=* ϕ_1_. The mixing results (*fl_a__mix*, *fl_b__mix*) are expressed as:

(14) $$\{\begin{array}{c} fl_{a}\_mix=fl_{a}\_\mathrm{mod}\cdot \cos\left(\omega_{0}t-\varphi_{ref1}\right)\\ fl_{b}\_mix=fl_{b}\_\mathrm{mod}\cdot \cos\left(\omega_{0}t-\varphi_{ref2}\right) \end{array}$$

Finally, the original fluorescence signal (*fl*_1_, *fl*_2_) can be obtained via low-pass filtering, as expressed in Equation (15). Both signals are resolved, but with a loss in amplitude [1/2cos(ϕ_1_ − ϕ_2_)]:

(15) $$\{\begin{array}{c} fl_{1}=\frac{1}{2}\cos(\varphi_{1}-\varphi_{2})C_{3}\exp[-{\left(t-t_{0}+\tau_{1}\right)}^{2}/(2\sigma^{2})]\\ fl_{2}=\frac{1}{2}\cos(\varphi_{2}-\varphi_{1})D_{3}\exp[-{\left(t-t_{0}+\tau_{2}\right)}^{2}/(2\sigma^{2})] \end{array}$$

## 3. Materials and Method

In this study, three steps are employed to evaluate and demonstrate PTDE. Mathematical modeling is first performed using MATLAB^®^, to simulate the PTDE theory and to verify the methodology. A variety of fictional fluorescence lifetime, peak, and crossover coefficients are employed. Secondly, fixed mammalian cells are stained with single-color reagents, so that the individual lifetime components of each fluorochrome can be acquired. Finally, experiments are conducted using fixed mammalian cells to test the fluorescence signal representation by discriminating between the fluorescence lifetimes of all samples.

### 3.1. Simulation of Time-Delay Estimation Fluorescence Measurements

The peak values and peak locations of the observed fluorescence pulse waveforms are influenced by *A*, *B*, Δ*t*_1_, Δ*t*_2_, and the crossover coefficients. Table 1 lists the different specifications and sample parameters used for the simulation.

### 3.2. Experiments Using Mammalian Cells

The samples used in this study were the Cyto-Trol Control Cells Kit (Ref. 6604248, Beckman Coulter Inc., Brea, CA, USA) and the fluorochrome-conjugated antibodies, CD3-FITC (Ref. A07746, Beckman Coulter Inc., Brea, CA, USA) and CD4-PC5 (Ref. A07752, Beckman Coulter Inc., Brea, CA, USA).

Cyto-Trol Control Cells are used for activity assessments of monoclonal antibodies via flow cytometry. These cells are a lyophilized preparation of human lymphocytes that exhibit surface antigens detectable with monoclonal antibodies. The cells are isolated from peripheral blood and express antigens representative of those found on normal lymphocytes. They are used with direct conjugation on monoclonal antibodies. The fluorochrome-conjugated antibodies facilitate the identification and numeration of cell populations expressing the CD3 and CD4 antigens present in human biological samples using flow cytometry. All fluorochrome-conjugated antibodies are excited at 488 nm with broad emission spectra that overlap from ~500 to 700 nm. The emission peaks of CD3-FITC and CD4-PC5 occur at 525 and 670 nm, respectively. Here, the flow cytometry measurements were acquired with a Gallios Flow Cytometer (Beckman Coulter Inc., Brea, CA, USA).

- The compensation tubes were stained with single-color reagents (CD3-FITC, CD4-PC5) in each fluorochrome and used to acquire the average fluorescence lifetime, with all these tubes being run in the same manner. The average fluorescence lifetime was taken as the individual lifetime component of FITC and PC5. The fluorescence pulse signals were detected, and the individual lifetime component was acquired using the MCZT and FICP techniques mentioned above.
- The verification tube was stained with two-color reagents simultaneously and used to ensure that the representation method based on the fluorescence PTDE is satisfactory for the samples and antibodies used in this work.

## 4. Results and Discussion

### 4.1. PTDE Simulation

**Figure 6:**
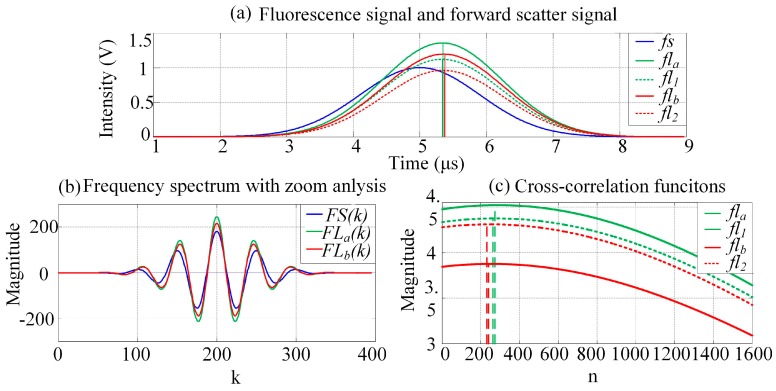
Simulation results at each stage during PTDE. (**a**) Waveforms of fluorescence and forward-scattered pulse signals. The colorized solid and dashed lines are the observed fluorescence signals after crossover and the original fluorescence signals, respectively; (**b**) The frequency spectra are zoom-analyzed 10 times (*N*_1_/*N* = 10 in Equation (6)) using MCZT; (**c**) Time domain cross-correlation functions calculated using FICP (*N*_2_/*N*_1_ = 10 in Equations (8) and (9)).

Sample results of the simulation output, for which the mathematical functions of each stage were modeled, are plotted in Figure 6a–c. For this example, the two fluorescence signals had simulated time delays of 0.33 and 0.35 μs, and the obtained peak intensities are 1.40 and 1.20 V, respectively. Figure 6a shows the original and observed fluorescence signals after crossover. The fluorescence-signal waveform peak values and peak locations are influenced by crossover. 

As shown in Figure 6a, *fl*_1_ is ahead of *fl*_2_. The crossover causes the location of the *fl_a_* peak to shift to the right, which increases the time delay between *fl_a_* and *fs* slightly. Further, *fl*_2_ lags behind *fl*_1_, and the location of the *fl_b_* peak shifts to the left as a result of the crossover. The time delay between *fl_b_* and *fs* decreases slightly. Figure 6b shows plots of the zoomed frequency spectra of the pulse signals (*fs*, *fl_a_*, and *fl_b_*). A zoom analysis of the frequency spectrum was then executed within the scope of a certain frequency and using the MCZT method, so as to acquire high-resolution frequency spectral information. Recall that this information was not limited by the pulse signal length or by the ADC sampling frequency. With considering the characteristic of frequency spectrum and the amount of calculations synthetically in this work, the values of *N*_1_ and *N*_2_ are set to be *N*_1_/*N* = 10 and *N*_2_/*N*_1_ = 10. Compared with the fast Fourier transform (FFT) result, less detailed information is lost and the frequency resolution of the spectrum obtained using the MCZT method is significantly higher; therefore, the corresponding cross-correlation frequency spectrum is smoother. Figure 6c shows plots of the cross-correlation functions calculated using the FICP algorithm. The time-delay resolution between *fs* and *fl_m_* in the time domain is increased to *N*_2_/*N*_1_ times with the same number of calculations.

Finally, the simulation results (see Table 1 for each parameter) are listed in Table 2. The expected peak values correspond to the signal intensities of the observed fluorescence, which form the corresponding original signals; that is, the expected peak values are *k*_11_ × *A* and *k*_22_ × *B*. The expected time delay values are Δ*T*_1_ and Δ*T*_2_. The peak-value errors and time delays were calculated using Equations (3) and (4). In this simulation, the crossover-induced peak-value errors ranged from 20.27% to 39.01%, whereas the crossover-induced time-delay estimation errors ranged from 0.31% to 3.46%. Thus, the influence of crossover on the peak values is significantly larger than that on the time-delay estimation. Further, the fluorescence lifetime, which is obtained from the time-delay estimation, is more suitable for the representation of spectrally overlapping fluorescence signals.

### 4.2. Time-Delay Estimation of Fluorescently Labeled Cells

The time delay between the *fl_a_*, *fl_b_*, and *fs* of the fluorescently labeled microspheres was estimated using the FICP algorithm, and the peak locations of these signals were acquired using a Gauss fitting algorithm, for comparison with the results of the proposed method. 

**Figure 7:**
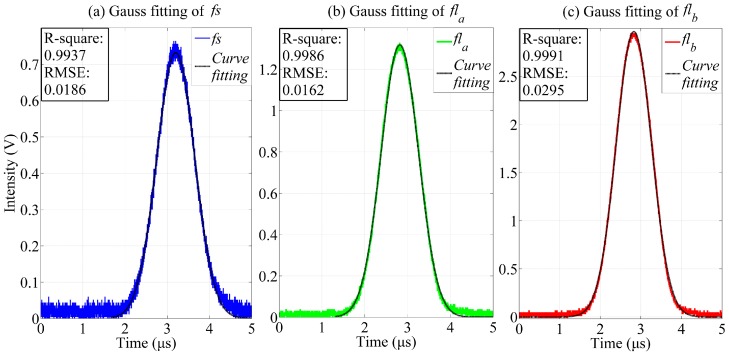
Gauss fitting results for pulse signals (*fs*, *fl_a_*, and *fl_b_*). (**a**) The blue and black curves are the pulse signal *fs* and the Gauss fitting result, respectively; (**b**) The green and black curves are the observed fluorescence pulse signal *fl_a_* and the Gauss fitting result, respectively; (**c**) The red and black curves are the observed fluorescence pulse signal *fl_b_* and the Gauss fitting result, respectively.

One series of observed *fs*, *fl_a_*, and *fl_b_* signals was taken as an example for the FICP feasibility analysis. The curves obtained after Gauss fitting are shown in Figure 7, where the colored curves are the observed signals and the black curves are the Gauss fitting results. 

The Gauss fitting was modeled by $ae^{-{\left(\frac{t-b}{c}\right)}^{2}}$ and the results were evaluated by calculating the root mean square error (RMSE) and the R-squared values. The RMSE is an estimate of the standard deviation of the random component in a data set, and was used to evaluate the deviation between the curve fitting result and the observed signal in this study. The RMSE is defined as:

(16) $$RMSE=\sqrt{\frac{1}{n}\sum_{i=1}^{n}{\left(X_{obs,i}-X_{fit,i}\right)}^{2}}$$

where *X_obs_* is the observed value, *X_fit_* is the curve fitting result, and *n* is the length of the observed signal, which is equal to that of the fitting result.

R-squared is the square of the correlation between the observed and predicted values. It is also known as the “square of the multiple correlation coefficient” and the “coefficient of multiple determination”. R-squared is defined as the ratio of the sum of the squares of the regression (SSR) and the total sum of squares (SST):

(17) $$R-squared=\frac{SSR}{SST}$$

SSR and SST are defined as:

(18) $$SSR=\frac{1}{n}\sum_{i=1}^{n}{\left(X_{fit,i}-{\bar{X}}_{obs,i}\right)}^{2}$$

(19) $$SST=\sum_{i=1}^{n}{\left(X_{obs,i}-{\bar{X}}_{obs,i}\right)}^{2}$$

where ${\bar{X}}_{obs}$ is the mean value of the observed signal. R-squared can take any value between 0 and 1, with a value closer to 1 indicating that a greater proportion of variance is accounted for by the model. Table 3 presents the Gauss fitting and FICP results. The RMSE of the Gauss fitting is less than 0.03 and the R-squared values are larger than 0.99. Therefore, the observed signals were represented perfectly by the Gauss fitting results. Meanwhile, the deviations in the time delays between *fl_a_* and *fs* and between *fl_b_* and *fs* are 3.4 ns and 1.6 ns, respectively.

Note that the time delays calculated above contain the time delays introduced by both the fluorochrome lifetimes and the electrical system. In the calibration process, Gaussian-shaped pulses having an average peak voltage of 2 V and an average pulse width ranging from 1 to 5 μs, in increments of 0.5 μs, were artificially created. Photoelectric detectors were configured to collect light signals from the LEDs. The processing methods were verified by introducing artificial fluorescence delays, which was achieved by setting the phase-shift values between the LED pulses to 0 ns. For each group of delays, 1000 events were collected and processed, and the mean values were taken as the calibration results.

Thus, 1000 serial pulse signals of fluorescently labeled microspheres were detected, and the time delays between *fl_a_*, *fl_b_*, and *fs* were calculated using Gauss fitting and the FICP method. Table 4 lists the statistical results of the time-delay estimation after calibration of the 1000 events. The mean values, standard deviations (SDs, $\sigma =\sqrt{\frac{1}{n-1}\sum_{i=1}^{n}{\left(x_{i}-\bar{x}\right)}^{2}}$) and relative standard deviations (RSDs, $\frac{\sigma }{\bar{x}}\;\times \;100\%$) were taken as indexes to evaluate the statistical properties of the fluorescence pulse signals obtained from the same type of fluorochrome.

As we can see, the mean values of each channel obtained via Gauss fitting and FICP are very close, and the SDs and RSDs determined for the FICP method values are significantly smaller than those for the Gauss fitting results. Therefore, the time delay can be estimated correctly using the FICP method, and the lifetimes (τ*_a_* and τ*_b_*) can be acquired with calibration. Meanwhile, the individual lifetime (τ_1_, τ_2_) components of each fluorochrome, which were acquired for cells labeled with one kind of fluorochrome and tested separately, are also listed in Table 4. It is apparent that τ*_a_* is smaller than τ_1_, whereas τ*_b_* is larger than τ_2_. Note that the deviations between τ*_a_*, τ*_b_* and τ_1_, τ_2_ are introduced by spectral overlap. Further, τ_1_ is longer than τ_2_, and the peak location of *fl_b_* moves right while that of *fl_a_* moves left, because of the influence of the spectral overlap. Therefore, the calculated lifetime of *fl_a_* is significantly smaller than that of *fl*_1_, whereas the calculated lifetime of *fl_b_* is considerably larger than that of *fl*_2_.

### 4.3. Fluorescence Signal Representation of Fluorescently Labeled Cells Based on PTDE

**Figure 8:**
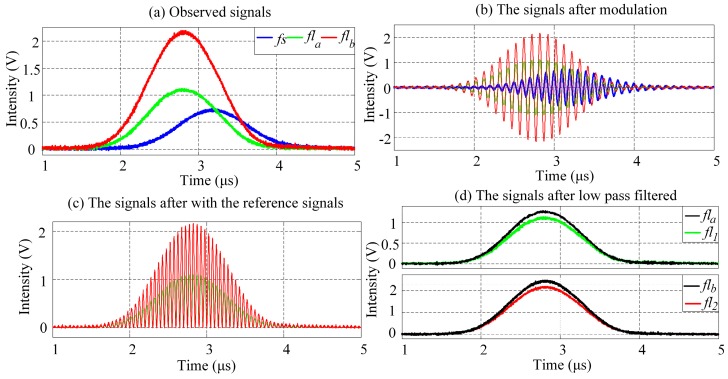
Results of PTDE-based fluorescence signal representation at each stage (see also Figure 3 for each stage of the digital signal processing). (**a**) Waveforms of forward-scattered pulse signal (*fs*) and observed fluorescence pulse signals (*fl_a_*, *fl_b_*) from a mixture of two spectrally overlapping signals; (**b**) *fl_a_* and *fl_b_* are modulated by a cosine function with different phase shifts (ϕ*_a_*, ϕ*_b_*); (**c**) The modulated fluorescence pulse signal is mixed with a reference signal having the same angular frequency as the modulating signal, after which low-pass filtering of the signals in (**c**) results in (**d**).

The width of observed cytometric pulses and the differences between lifetimes τ_1_ and τ_2_, together with the amount of calculations are considered collectively, the modulating frequency is set to be 10 MHz (ω_0_ = 10 MHz) for the analysis of crossover influence eliminating. Figure 8a–d is a panel of waveforms that exemplify the step-wise fluorescence signal representation of fluorescently labeled cells based on PTDE. The τ*_a_* and τ*_b_* lifetimes were used to artificially configure the signals after modulation with Equation (12), as shown in Figure 8b. In the figures, each lifetime is reflected and represented with the phase shift introduced by the lifetime. The signals after modulation are mixed with reference signals so as to eliminate the crossover influence, as shown in Figure 8c. Finally, the original fluorescence signals are acquired using a low-pass filter.

As Figure 8 reveals, the PTDE-based fluorescence signal representation method for the fluorescently labeled cells successfully and effectively eliminates the influence of the spectral overlap. Moreover, the modulation, mixing with reference signals, and low-pass filtering are performed using a digital signal processing method, thereby obviating the need for a high-speed analog device and complex circuit system. Further, the flexibility of the comprehensive method developed in this work is significantly higher than that of existing methods. For example, the modulating frequency can be significantly higher than that achievable for the analog method. Selected sample results for the mean phase shift at different modulating frequencies are listed in Table 5.

**Figure 9:**
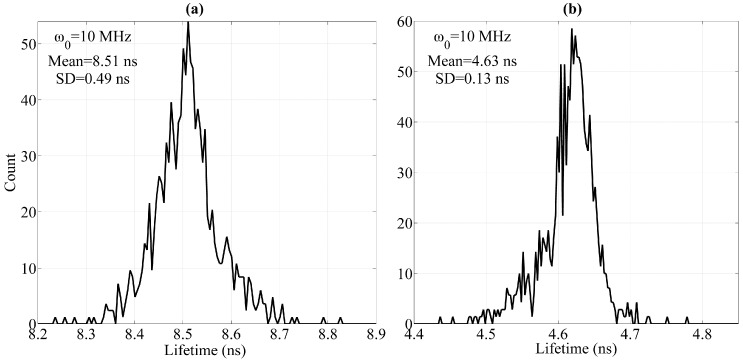
Lifetime histograms of fluorescence pulse signals at 10-MHz modulating frequency. (**a**) Histograms of (**a**) *fl*_1_ and (**b**) *fl*_2_ lifetimes (SD: Standard deviation).

Finally, the lifetime histograms of the fluorescence pulse signal (ω_0_ = 10 MHz) are provided in Figure 9. Limited to the experimental conditions employed in this work, the mean values of the calculated individual lifetimes of each fluorochrome are 8.51 ns (SD = 0.49 ns) and 4.63 ns (SD = 0.13 ns). The calculated individual lifetimes after the influence of spectral overlap is eliminated are close to the individual lifetimes of each fluorochrome (for the examined cells stained with single-color fluorochrome). Thus, the influence of spectral overlap can be eliminated effectively using the comprehensive method presented in this work.

## 5. Conclusions

In this paper, a method through which fluorescence pulse signals with spectral overlapping can be represented was proposed. The MZCT algorithm was introduced for zoom analysis of the frequency spectra of forward-scattered and multicolor fluorescence pulse signals, to prevent information loss through the fence effect. Then, the cross-correlation function was calculated using FICP, so as to increase the time-domain resolution of the fluorescence pulse time-delay estimation (PTDE). Subsequently, after modulation, signals were created with a digital signal processing method to convert the lifetimes to phase shifts, and reference signals with specific phase shifts were introduced to eliminate spectral overlap. The proposed method using digital signal processing offers a more flexible approach than existing methods and, also, complex analog circuits are not required. In conclusion, a PTDE-based method for representing fluorescently labeled cells is recommended for fluorescence lifetime analysis via flow cytometry, especially for two-color analysis scenarios adversely affected by extensive spectral overlap. On the other hand, in the proposed method, the autofluorescence overlap are simplified by regarding them as part of the emission fluorescence. Thus, the accuracy with which the spectral overlap influence is eliminated will be improved when the autofluorescence can be distinguished and eliminated. Further, the method proposed in this paper is not suitable for multi-exponential decay or cases in which more than two colors are stained simultaneously. Thus, the focus of future work could be the representation of spectrally overlapping signals in both cases using simple algorithms and hardware.

## Figures and Tables

**Table 1:** Example parameters used in simulation.

No.	*A* (V)	*B* (V)	Δ*t*_1_ (μs)	Δ*t*_2_ (μs)	*k*_11_	*k*_12_	*k*_21_	*k*_22_
1	1.40	1.20	0.33	0.35	0.80	0.15	0.15	0.80
2	1.10	1.20	0.33	0.35	0.80	0.15	0.15	0.80
3	1.40	1.20	0.30	0.40	0.80	0.15	0.15	0.80
4	1.10	1.20	0.30	0.40	0.80	0.15	0.15	0.80
5	1.40	1.20	0.33	0.35	0.80	0.10	0.10	0.80
6	1.40	1.20	0.33	0.35	0.75	0.15	0.10	0.75

**Table 2:** Example simulation results.

No.		*fl_a_*		*fl_b_*	
		Peak	Time-Delay	Peak	Time-Delay
1	Expected values	1.12 V	0.33 μs	0.96 V	0.35 μs
	Errors (%)	20.95	1.70	39.01	1.42
2	Expected values	0.88 V	0.33 μs	0.96 V	0.35 μs
	Errors (%)	28.37	3.46	38.99	2.32
3	Expected values	1.12 V	0.30 μs	0.96 V	0.40 μs
	Errors (%)	20.79	1.84	38.82	−0.93
4	Expected values	0.88 V	0.30 μs	0.96 V	0.40 μs
	Errors (%)	28.15	2.92	38.82	1.12
5	Expected values	1.12 V	0.33 μs	0.96 V	0.35 μs
	Errors (%)	20.27	1.12	25.88	−0.78
6	Expected values	1.05 V	0.33 μs	0.90 V	0.35 μs
	Errors (%)	27.32	1.58	33.68	0.31

**Table 3:** Gauss fitting and FICP results (*N*_1_/*N* = 10, *N*_2_/*N*_1_ = 10).

	Gauss Fitting				FICP
	Peak Location (ns)	RMSE	R-Square	Time-Delay (ns)	Time-Delay (ns)
*fs*	5870	0.0186	0.9937	-	-
*fl_a_*	5108	0.0162	0.9986	762	758.6
*fl_b_*	5120	0.0295	0.9991	750	748.4

**Table 4:** Statistical results of time-delay estimation after calibration.

	Gauss Fitting			FICP		
	Mean (ns)	SD (ns)	RSD (%)	Mean (ns)	SD (ns)	RSD (%)
τ*_a_*	8.35	0.38	4.44	8.28	0.32	3.77
τ*_b_*	4.90	0.15	3.26	4.86	0.11	2.36
τ_1_	8.56	0.76	8.50	8.48	0.58	6.55
τ_2_	4.58	0.24	5.36	4.67	0.17	3.84

**Table 5:** Mean values of phase shifts at different modulating frequencies.

	ω_0_ (MHz)					
	1	2	5	10	20	50
ϕ*_a_* (°)	83.13	86.54	88.61	89.29	89.65	89.86
ϕ*_b_* (°)	78.95	84.37	87.70	88.84	89.41	89.77

## Abbreviations

The following abbreviations are used in this manuscript:

PTDE	Pulse Time-Delay Estimation
ADC	Analog-to-Digital Converter
MCZT	Modified Chirp Z-Transform
FICP	Fine Interpolation of Correlation Peak
RMSE	Root Mean Square Error
SSR	Sum of Squares of the Regression
SST	Total Sum of Squares
SD	Standard Deviation
RSD	Relative Standard Deviation
